# Section on Obstetrics, Texas State Medical Association

**Published:** 1901-03

**Authors:** William Keiller, O. L. Norsworthy

**Affiliations:** Galveston, Chairman; Houston, Secretary


### Section on Obstetrics, Texas State Medical Association.

DEAR DOCTOR: The Texas State Medical Association will meet in Galveston on Tuesday, April 23, 1901.

As chairman and secretary of the section on obstetrics we desire to remind you that though the subject is one full of interest to every physician, the success of the section will depend upon the individual efforts of the members of the Association.

We feel sure that you have only to look back over the experience of the past year to find some case or cases, or some phase of the subject which has specially interested you, and we urge that you furnish our section with a paper.

To be in time for the printer titles of papers should be in the hands of the secretary not later than April 10, 1901.

Fraternally yours,
WILLIAM KEILLER, Galveston, Chairman,
O. L. NORSWORTHY, Houston, Secretary.

